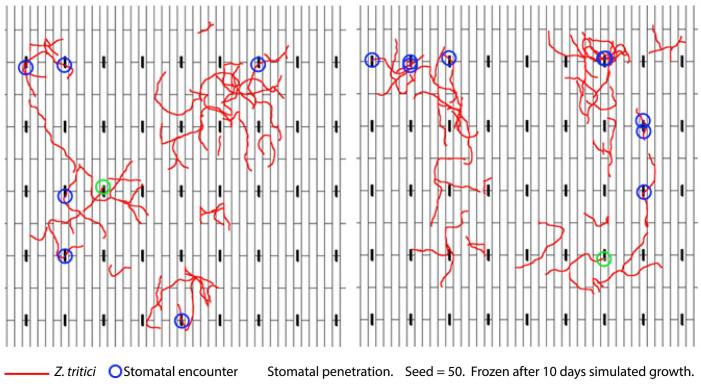# A role for random, humidity-dependent epiphytic growth prior to invasion of wheat by *Zymoseptoria tritici*

**DOI:** 10.1016/j.fgb.2017.07.002

**Published:** 2017-09

**Authors:** Helen N. Fones, Chris J. Eyles, William Kay, Josh Cowper, Sarah J. Gurr

**Affiliations:** a Geoffrey Pope Building, University of Exeter, Stocker Road, Exeter EX4 4QD, UK; b Rothamsted Research, North Wyke, Okehampton, Devon EX20 2SB, UK; c Donder’s Hon Chair, University of Utrecht, Padualaan 8, 3584 CH Utrecht, The Netherlands

**Keywords:** Crop pathogens, Epiphytic growth, Food security, *Mycosphaerella graminicola*, Humidity, Septoria leaf blotch, Wheat, *Zymoseptoria tritici*

## Abstract

*Zymoseptoria tritici* causes Septoria leaf blotch of wheat. The prevailing paradigm of the *Z. tritici*-wheat interaction assumes fungal ingress through stomata within 24–48 h, followed by days of symptomless infection. This is extrapolated from studies testing the mode of fungal ingress under optimal infection conditions. Here, we explicitly assess the timing of entry, using GFP-tagged *Z. tritici*. We show that early entry is comparatively rare, and extended epiphytic growth possible. We test the hypotheses that our data diverge from earlier studies due to: i. random ingress of *Z. tritici* into the leaf, with some early entry events; ii. previous reliance upon fungal stains, combined with poor attachment of *Z. tritici* to the leaf, leading to increased likelihood of observing internal versus external growth, compared to using GFP; iii. use of exceptionally high humidity to promote entry in previous studies. We combine computer simulation of leaf-surface growth with thousands of *in planta* observations to demonstrate that while spores germinate rapidly on the leaf, over 95% of fungi remain epiphytic, growing randomly over the leaf for ten days or more. We show that epiphytic fungi are easily detached from leaves by rinsing and that humidity promotes epiphytic growth, increasing infection rates. Together, these results explain why epiphytic growth has been dismissed and early ingress assumed. The prolonged epiphytic phase should inform studies of pathogenicity and virulence mutants, disease control strategies, and interpretation of the observed low *in planta* growth, metabolic quiescence and evasion of plant defences by *Zymoseptoria* during symptomless infection.

## Highlights

• *Zymoseptoria tritici* is capable of over ten days of epiphytic growth on wheat leaves.
• Epiphytic growth and stomatal entry data are consistent with a random growth model.
• *Z. tritici* is easily dislodged from the leaf surface, by even gentle misting.
• Humidity promotes the hyphal growth form and facilitates rapid entry.
• Interpretation of “-omics” data must account for epiphytic growth, where appropriate.

## 1 Introduction

*Zymoseptoria tritici* is the most serious fungal pathogen of wheat in Europe, causing losses of around 5–10% in the UK, even when resistant wheat varieties are grown and fungicides applied (Fones and Gurr, 2015). Considering its importance, understanding of this fungus has, until recently, been comparatively limited (Solomon, 2017). This knowledge gap is being bridged (see Steinberg, 2015, Talbot, 2015, Zhong et al., 2017), but uncertainties regarding aspects of this pathogen’s lifestyle and interaction with its host remain (Sánchez-Vallet et al., 2015). These uncertainties include questions over (a) the timing of fungal entry into the wheat leaf, (b) whether the fungus actively seeks stomata and, (c) the feeding and defence evasion mechanisms of the fungus prior to the onset of symptoms in the host. Further, most investigators to date have used very high inoculum densities, following the 1–2 × 10^7^ cfu/ml inoculum used in initial studies by Eyal et al. (1985) (e.g. Kema et al., 1996, Duncan and Howard, 2000), or choosing to increase the inoculum density further (eg to 10^8^ cfu/ml, Zuckerman et al., 1997) and it is not clear whether this impacts either host resistance or pathogen infection behaviour.

Sexual (ascospores) and asexual spores (conidia, or pycnidiospores) of *Z. tritici* germinate on wheat leaves, producing hyphae which enter the leaf *via* stomata (Kema et al., 1996, Duncan and Howard, 2000, Keon et al., 2007). Some authors claim that *Z. tritici* senses and grows toward stomata (Duncan and Howard, 2000, Cousin et al., 2006), while others have pointed to the fact that hyphae often cross stomata without entering the leaf as evidence against such directed growth (Kema et al., 1996, Shetty et al., 2003). The time required for penetration has variously been reported to be from twelve hours to three days (Kema et al., 1996, Duncan and Howard, 2000, Cousin et al., 2006, Keon et al., 2007). Penetration is followed by seven to ten days of symptomless infection (Kema et al., 1996, Keon et al., 2007, Orton et al., 2011, Lee et al., 2014). This symptomless phase can be prolonged under certain field conditions or with particular strain/variety combinations. During this time, *Z. tritici* grows very slowly. Whilst a visible increase in mycelium was reported in one study, invasive hyphae were few and close to the stomata through which ingress had occurred (Kema et al., 1996). ELISA assays did not detect fungal antigen until 7 days post inoculation (Kema et al., 1996), neither did RNAseq or metabolomics provide evidence for the presence of *Z. tritici* in infected wheat samples in this period (Rudd et al., 2015). No feeding structures have been observed, with *Z. tritici* remaining strictly apoplastic throughout infection (Kema et al., 1996, Duncan and Howard, 2000, Keon et al., 2007, Orton et al., 2011, Lee et al., 2015). The mechanism of nutrient acquisition by the fungus during the symptomless phase remains unclear, with controversy as to whether this fungus is a ‘stealthy’ hemibiotroph or a latent necrotroph (Marshall et al., 2011, Brunner et al., 2013, Goodwin et al., 2011, Sánchez-Vallet et al., 2015).

Recent genomic and transcriptomic studies provide clues about the nutrients used by *Z. tritici*. The genome of *Z. tritici* encodes few CAZy (Carbohydrate-Active Enzyme) family proteins, but an expanded repertoire of peptidase and alpha-amylase enzymes, compared to other ascomycete cereal pathogens. This suggests that *Z. tritici* derives nutrients differently from these fungi (Goodwin et al., 2011). *Z. tritici* expresses lipases, cutinases, and fatty acid metabolism enzymes during the symptomless phase (Keon et al., 2007, Rudd et al., 2015, Palma-Guerrero et al., 2016). This may indicate that *Z. tritici* is not greatly dependent on the host during this phase, but relying mainly upon stored energy, possibly supplemented by cuticular waxes (Rudd et al., 2015, Kettles and Kanyuka, 2016, Yang and Yin, 2016).

The experiments upon which the early-entry paradigm is based (e.g. Kema et al., 1996, Duncan and Howard, 2000, Cousin et al., 2006) were conducted to assess the mode, rather than timing, of fungal entry. These experiments used conditions designed to promote infection, including extended misting of leaves after inoculation, low light intensity or darkness, and extremely high inoculum densities. Here, we aim to determine, instead, the average time required for fungal leaf entry, and seek to determine whether epiphytic fungi have a role in disease by testing the effect of surface acting *vs.* systemic fungicides when applied at various times after inoculation. We use a GFP-tag in the model *Z. tritici* strain, IPO323, avoiding the need for staining procedures that may dislodge surface fungi; we also explicitly test the hypothesis that fungi may be easily detached from the leaf surface. We investigate the behaviour of the pathogen before penetration, to determine whether stomata are sought or encountered at random. Finally, we explore the effect of different humidity regimes upon this process, to determine whether changes in humidity affect the process of finding and penetrating stomata. Through these investigations, we endeavour to characterise more fully the symptomless stage of the wheat-*Z. tritici* interaction. We believe that clarification of the timing of entry, and the proportion of live fungus that has entered the leaf at a given time point, will be useful for the interpretation of transcriptomic and other results which provide data averaged across all live fungus in a sample. Similarly, we believe that elucidation of whether fungus growing on the leaf surface is able to seek stomata will facilitate the interpretation of data concerning its survival, gene expression, and pathogenicity. To fully utilise such knowledge, an understanding of the interaction between internal *vs*. external growth, penetration events, and known important environmental factors, such as humidity, is also needed.

## 2 Methods

### 2.1 Pathogen strains and culture conditions

A cytoplasmic GFP-tagged variant of the Dutch model strain of *Z. tritici*, IPO323 (Kema and van Silfhout, 1997), was used throughout this work. This strain carries the *gfp* gene integrated into the *Z. tritici* genome at the *sid1* locus, using the methods and vectors described in Kilaru et al. (2015). A second IPO323 variant was used in which the GFP tag primarily labels the plasma membrane (Kilaru et al., 2017). In this variant, the *gfp* gene was integrated into the IPO323 genome at random*.* The strains were kindly provided by Dr. S Kilaru and Prof G Steinberg. *Z. tritici* was stored in glycerol at −80 °C until required, then streaked onto YPD agar and incubated at 18 °C.

### 2.2 Plants and growth conditions

The wheat variety used throughout was Galaxie (Fenaco, Bern, Switzerland), with the exception of the data shown in Figs. S3–S5, where Consort (RAGT, Cambridge, UK) was additionally used. Both varieties are susceptible to the strain IPO323. Seeds were sown on damp John Innes No. 2 compost and kept under plastic cloches in a growth chamber at 20 °C, 80% RH and with a 12 h light/dark cycle until germination (4 days). Cloches were then removed and other conditions maintained for 10 days.

### 2.3 Inoculations

**Fig. 1:**
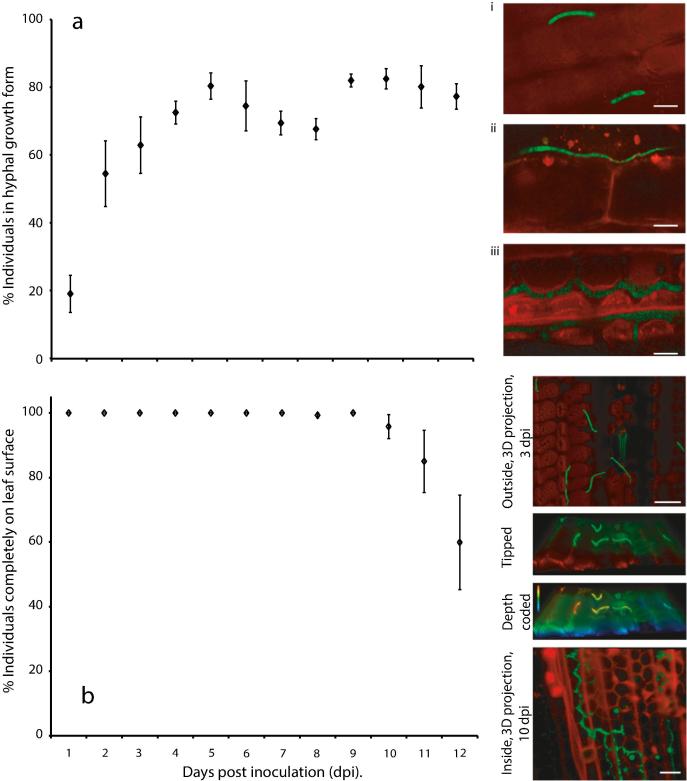
*Z. tritici* during the first 12 days following inoculation of IPO323 onto Galaxie wheat leaves. Fully expanded leaves of 14 days old Galaxie wheat were inoculated with *Z. tritici* at 10^5^ cfu/ml. Leaf samples were viewed using confocal microscopy and a minimum of thirty fungal individuals were photographed and scored for hyphal growth and for penetration of the leaf on days one to twelve. IPO323 expressing cytoplasmic GFP (bright green) are seen against autofluorescence from wheat chlorophyll (red). a. Proportion of individuals showing hyphal growth each day. Insets: i. Example of non-hyphal individuals; ii. hyphal growth (external) iii. hyphal growth (internal). Scale bars represent 10 μM. b. Proportion of individuals growing exclusively on the leaf surface on each day. Insets, top to bottom: example of external fungi shown as a 3D projection of a confocal z-stack; same 3D projection, tipped away from the viewer and with green channel boosted to make green autofluorescence (from plant cell walls) visible, outlining the leaf surface and showing that the fungi sit on the surface; same image depth coded red-blue (top-bottom) along the z-axis, showing the fungi in a redder (higher) shade than the nearby plant cells; example of a 3D projection of a z-stack showing internal fungus only, plant cell walls counterstained with propidium iodide, this image only. Data are means of five independent experiments; error bars show SE. Scale bars represent 25 μM. (For interpretation of the references to colour in this figure legend, the reader is referred to the web version of this article.)

**Fig. 2:**
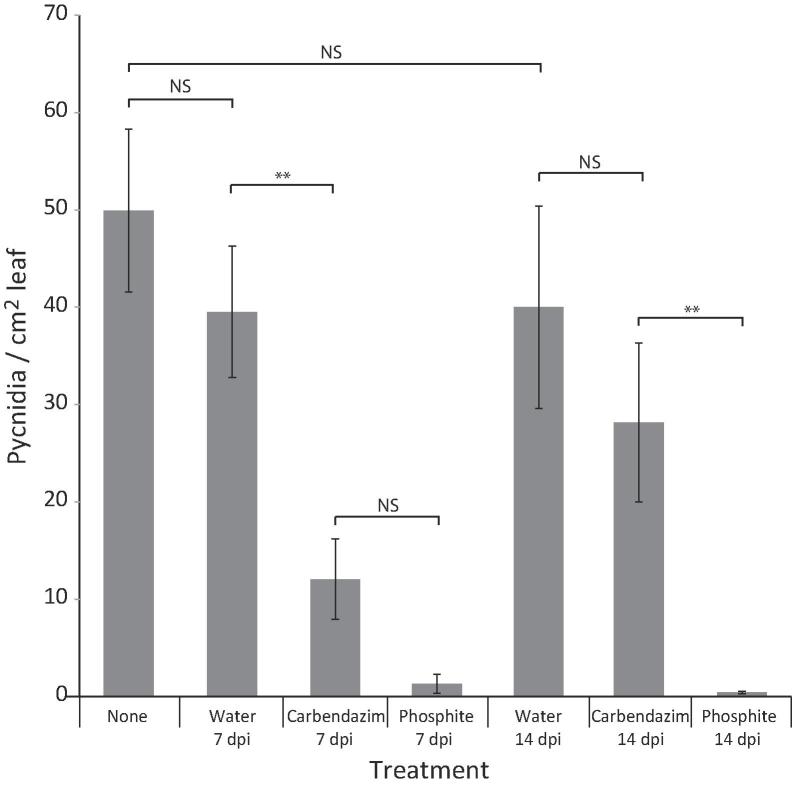
*Z. tritici* remains vulnerable to surface acting fungicide at 7 dpi. The first and second true leaves of twenty 21 day old Consort plants were inoculated with *Z. tritici* at 10^5^ cfu/ml*.* Plants were sprayed with water, carbendazim or phosphite at 7 or 14 dpi, or left untreated. A minimum of six plants were used per treatment. Pycnidia were enumerated at 28 dpi. Values are means and error bars represent SE. Asterisks indicate significant differences from control (Bonferroni corrected *t-*tests). This experiment was repeated with similar results.

**Fig. 3:**
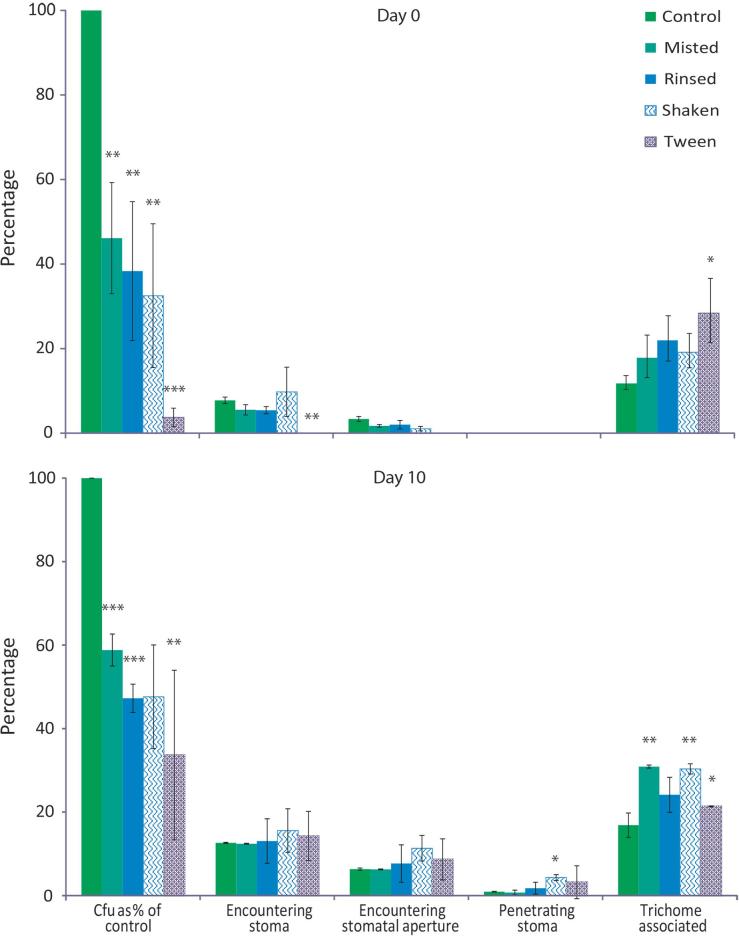
*Z. tritici* is easily dislodged from the wheat leaf. Leaves were inoculated with *Z. tritici* at 10^7^ cfu/ml and allowed to dry at RT for 1 h. Leaves were either: sampled immediately (controls); gently misted with water to runoff; rinsed by placing in a 15 ml tube half filled with water and inverting ten times; or vigorously shaken in a half-filled 15 ml tube of water or 0.1% (v/v) Tween. Inoculated leaves were visualised using confocal microscopy and all individuals in a given area enumerated and their location scored. Values are means of 2 or 3 independent experiments, each consisting of 5 biological replicates each including 3 samples; control n = 30–200. Error bars represent SE. Asterisks indicate significant differences from control (Bonferroni corrected *t*-tests). Fungi were scored as encountering a stoma if any part of the fungus touched or crossed the guard cells. Penetration was scored if fungus was visible in the substomatal aperture. Trichome association indicates that some part of the fungus grew across a trichome. Cfu counts are given as a percentage of the number seen on control (unwashed) samples (first set of bars). Other values represent the percentage of fungi within each treatment in the indicated location.

**Fig. 4:**
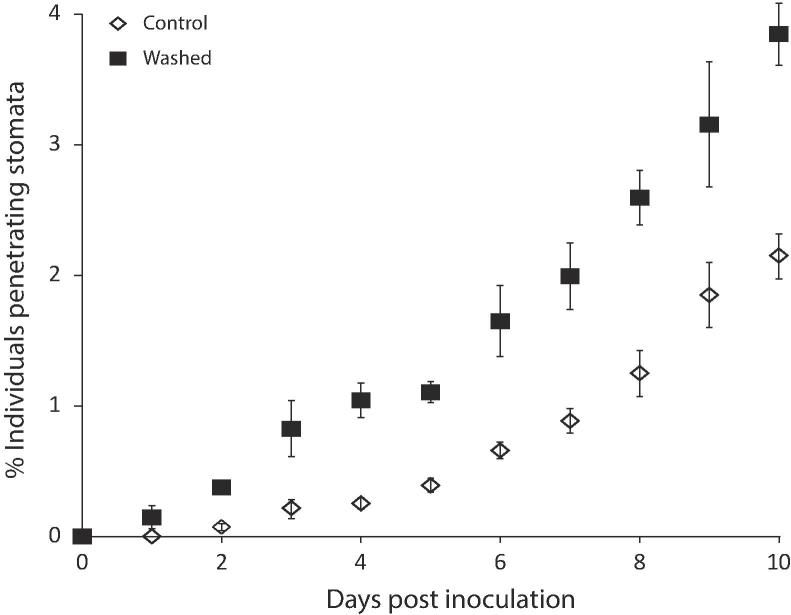
The percentage of *Z. tritici* individuals penetrating stomata increases with time and with rinsing of sampled leaves, and is not zero even at 1 dpi. Inoculated leaves (10^6^ cfu/ml) were sampled every day from 0–10 dpi and visualised at 20× magnification using a confocal microscope. Individuals of *Z. tritici* were scored for penetration of stomata on rinsed and control leaf samples. Results show average percentages from three randomly chosen leaf pieces. Number of fungi scored on each ranged from 271 to 1593, with a mean sample size of 840. Both datasets can be described by an exponential curve (R^2^ > 95%). Data are means of three independent experiments and error bars represent SE. A two-way ANOVA showed a significant effect of both dpi and washing (P < 0.001).

**Fig. 5:**
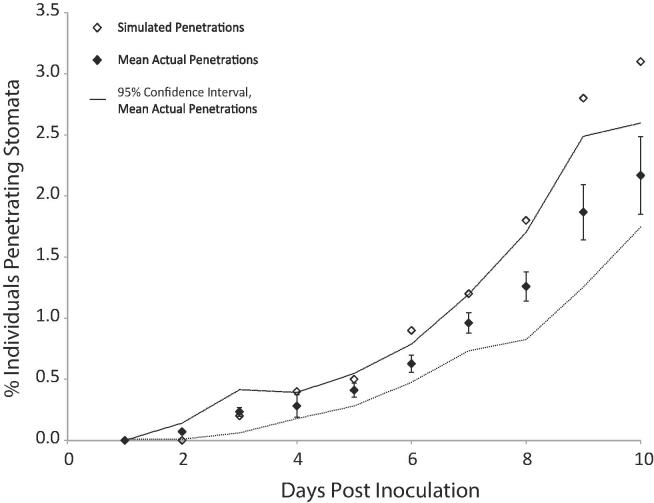
Occurrence of simulated and actual stomatal penetration events over 10 days. Fungal growth was modelled assuming random growth. Growth rate, probability of changing direction, branching, and following plant cell walls were estimated from images gathered for experiments described in Fig. 1, Fig. 2. Probability of penetrating encountered stomata was from published data. Simulated data are represented by open symbols. Closed symbols represent the means of three independent experiments in which *Z. tritici* was scored for penetration of stomata on leaf samples each day (see Fig. 4); error bars show SE and lines indicate upper and lower bounds of 95% confidence intervals of these means. Bonferroni corrected *t-*tests, using the simulated data point as a estimate of the population mean, were carried out where the simulated data point falls outside of these confidence intervals, but no significant results were obtained (6 dpi, P = 0.16; 8 dpi, P = 0.28; 9 dpi, P = 0.20 and 10 dpi, P = 0.10).

**Fig. 6:**
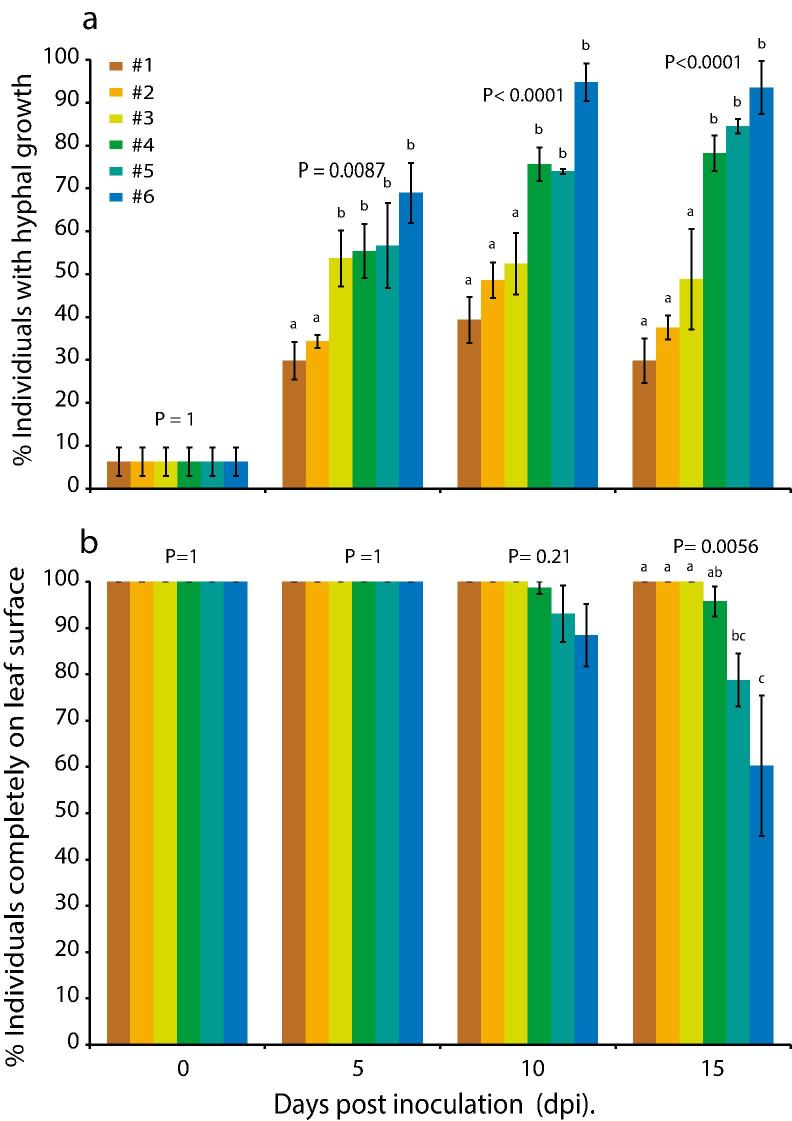
Effect of humidity upon hyphal emergence and leaf penetration over time. Galaxie wheat plants were inoculated with 10^6^ cfu/ml suspensions of *Z. tritici* and maintained under six different humidity regimes (#1 – lowest, #6 – highest). Inoculated leaves were visualised at 0, 5, 10 and 15 dpi and fungi scored for hyphal growth and leaf penetration. a. Hyphal emergence, b. penetration. Data are means of three independent experiments; error bars show SE. Within each time-point, results that are not significantly different are marked with the same letter (ANOVA with Tukey’s simultaneous comparisons; P-values above graphs).

**Fig. 7:**
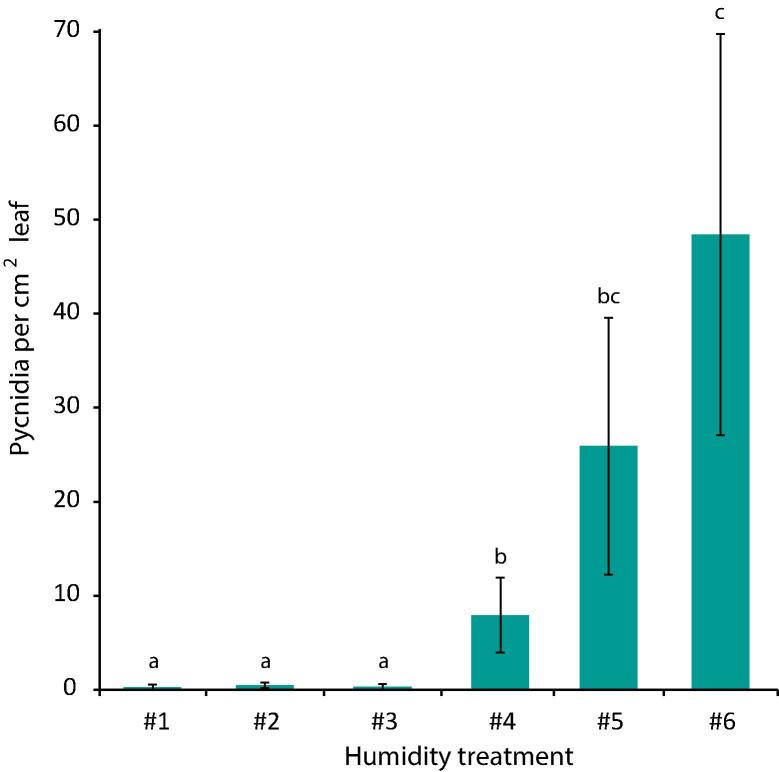
Effect of humidity pycnidiation by *Z. tritici* IPO323. Galaxie wheat plants were inoculated with 10^6^ cfu/ml suspensions of *Z. tritici* and maintained under six different humidity regimes (#1 – lowest to #6 – highest). Inoculated leaves were examined at 21 dpi and pycnidia enumerated. Data are means of three independent experiments; error bars show SE. Different letters indicate significant differences between results (ANOVAs with Tukey’s simultaneous comparisons).

**Fig. 8:**
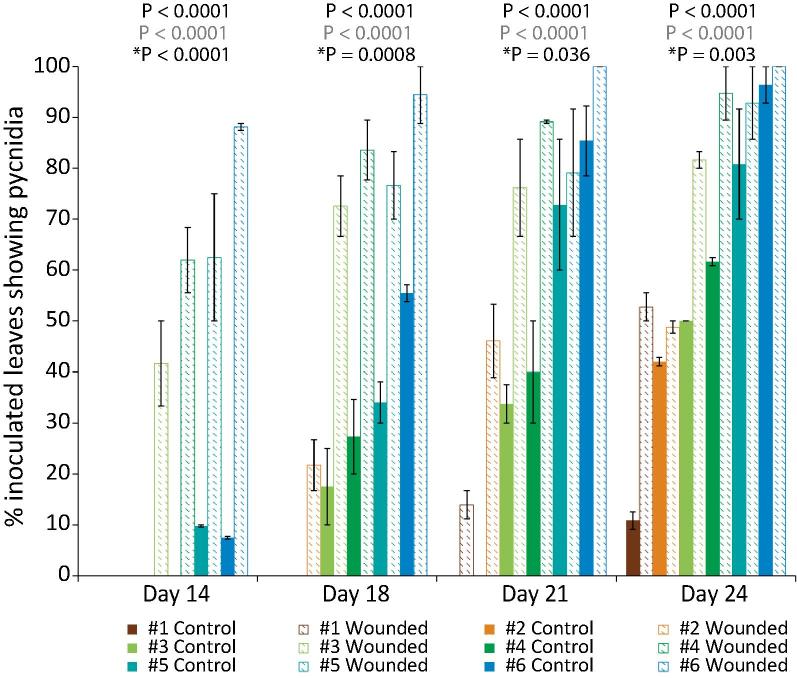
Effect of wounding of host leaves on pycnidiation by *Z. tritici* IPO323 under six humidity regimes. Leaves were gently abraded prior to inoculation with 10^6^ cfu/ml suspensions of *Z. tritici.* Control leaves were inoculated, but not abraded. Plants were then maintained under six different humidity regimes (#1 – lowest; #6 – highest). Inoculated leaves were examined from 14 dpi onwards, and the percentage of inoculated leaves bearing pycnidia determined on each day. Data are means of two independent experiments; error bars show SE. Within each time point, two-way ANOVAs were carried out. In each case, humidity, wounding, and the interaction between these treatments were all significant predictors of pycnidiation (P-values above graphs; black text = humidity, grey text = wounding, ^*^ = interaction term).

Fourteen day-old plants were used for inoculations. Fully expanded leaves were marked at the base and a spore suspension applied using a paintbrush. For most experiments, spore suspensions were created from three day old streak plates of *Z. tritici* by vortexing a small quantity of yeast-like growth in 0.1% (v/v) Tween. This suspension was passed through two layers of sterile Miracloth to remove hyphal or large branching structures and diluted to give a suspension of 10^5^–10^7^ spores/ml depending upon application (Fig. 1, Fig. 2: 10^5^cfu/ml; Fig. 4, Fig. 5, Fig. 6, Fig. 7, Fig. 8: 10^6^ cfu/ml; Fig. 3: 10^7^ cfu/ml). Inoculated plants were returned to the growth chamber until 21 days post inoculation. To ensure that the results obtained are not artefacts caused by use of inoculum produced *in vitro*, this experiment was repeated using pycnidiospores produced by cytoplasmic GFP-tagged IPO323 in Galaxie wheat. To obtain pycnidiospores, plants were inoculated in the same manner and leaves bearing pycnidia harvested at 21 dpi. Leaves were transferred to falcon tubes containing 0.1% (v/v) Tween and agitated to suspend spores. Spore suspensions were filtered, diluted and used to inoculate plants as for yeast-like growth. To ensure that the results are not artefacts caused by the GFP-tag, the experiment was repeated using a second strain of IPO323 in which a different subcellular compartment–the plasma membrane–was labelled and the *gfp* gene integrated into the genome at random rather than targeted to the *sid1* locus (Kilaru et al., 2017). Finally, to be certain that the results seen are not unique to the Galaxie-IPO323 interaction, a second susceptible wheat variety, Consort, was used in an experimental repeat, using both GFP-tagged strains.

### 2.4 Confocal microscopy

Confocal images were obtained using a Leica SP8 with LAS-X software and 40× oil immersion lens, 60× water immersion lens or 20× dry lens. An argon laser with emission at 500 nm was used to excite GFP fluorescence and chloroplast autofluorescence, detected at 510–530 and 600–630 nm, respectively. Samples of infected leaf material were mounted in water and fields of view selected randomly. Z-stacks were taken which encompassed fungi entirely. Fungi were scored for inside/outside leaves by looking (a) through stacks for any green fluorescent fungi inside leaves; (b) at 3D projections of stacks, tipping the projection to obtain views from various angles and (c) by use of the Leica LAS-X software’s depth coding function. Fungi were scored as internal if any part of an individual was so. In the context of the work presented here, the term ‘individual’ is taken to mean any contiguous fungal unit, which may be a single yeast-like cell (arising from budding growth), a branching macroconidium of any size, or a hyphal network. It may also be a mixture of macroconidial-type and hyphal-type cells. All of these types of individual can be observed on leaves initially inoculated with either filtered ‘yeast-like’ growth from a plate or with pycnidiospores raised *in planta.* Cells within an individual are interconnected and can be assumed to share nutrients and other signals. Time-courses of confocal microscopy were halted at 12 dpi, as after this point and individuals could not be easily distinguished. Hyphal growth was scored if any portion of an individual exhibited the hyphal growth form (characterised by long, narrow cells readily distinguished from the shorter, wider cells of both yeast-like growth and pycnidiospores). Five independent experiments were carried out to score fungal individuals for hyphal growth and for location at the inside/outside of the leaf. In each, a minimum of thirty fungal individuals were scored each day, from at least three randomly selected fields of view on each of three randomly selected leaf pieces.

### 2.5 Fungicide experiments

For fungicide assays, sprays were carried out at 7 or 14 dpi, using an Iwata Revolution SAR airbrush. Fungicide solutions were adjusted to pH 6, mixed with 0.01% (v/v) Silwet L-77 (Momentive Specialty Chemicals, UK), and sprayed on both surfaces of the leaves at a rate of 1 ml per cell of a 24-cell tray (2 plants per cell, avoiding run off). Phosphite (prepared from phosphorous acid; Sigma, UK), an antifungal which causes plant defence elicitation (Liu et al., 2016), was used at a concentration of 100 mM; carbendazim (Sigma, UK), a benzimidazole fungicide which is active on the leaf surface (FAO, 1992) was used at a concentration of 100 μM. Controls consisted of either no fungicide application or mock treatment with water only, again avoiding runoff. To quantify disease, leaves were removed from plants 28 days after inoculation, and taped, abaxial side up, onto 3mm white perspex sheets and weighed down between dry tissue papers for 24 h. Leaves were scanned at high resolution and pycnidia number and leaf area measured using a bespoke ImageJ (Abramoff et al., 2004) script. Two independent repeats of this experiment were carried out.

### 2.6 Washing experiments

Plants were inoculated and then returned to the growth chamber as normal. However, randomly selected plants, instead of being returned to the growth chamber, were allowed to dry for 1 h. Washing treatments were then immediately applied to the leaves of these plants: ‘Control’ leaf samples were not washed; ‘Misted’ leaf samples were sprayed with a fine, gentle spray of water using an atomiser and hand pump until run-off; ‘Rinsed’ sampled were placed in 15 ml falcon tubes containing 7.5 ml water and inverted ten times and ‘Shaken’ samples were treated in the same manner but inversion replaced by 30 s vigorous shaking by hand. For ‘Tween’ samples, the same procedure was followed as for ‘Shaken’ samples, but the water replaced by a 0.1% (v/v) Tween solution. Five randomly chosen leaf samples from each of these washing treatments were then immediately mounted for microscopic analysis. The number of spores in each of three randomly selected fields of view was then enumerated for each sample, and their location with respect to leaf features such as stomata and trichomes scored. On selected subsequent days, some of the remaining plants, that had been returned to in the growth chamber following initial inoculation, and had never been exposed to washing treatment, were randomly selected and washing treatments applied. This allowed the ease with which the fungus was dislodged from the leaf to be studied after normal growth *in planta*, as well as immediately following inoculation. The whole experiment was repeated on three independent occasions.

### 2.7 Stomatal penetration searches

Daily searches for stomatal penetration events were carried out on control and ‘Rinsed’ leaf samples. Using 20× magnification, large numbers of fungi were viewed on the leaf surface: the mean number of fungal individuals viewed per sample was 840. Each sample consisted of five random fields of view from each of three randomly chosen leaf pieces. Any fungi that encountered stomata were also viewed at 40× or 60× magnification and z-stacks viewed to obtain accurate scoring of penetration events.

### 2.8 Simulation of fungal growth *in silico*

Fungal growth was simulated *in silico* on a simulated leaf surface whose cell sizes, stomatal sizes and stomatal spacing were based on measurements made on Galaxie wheat leaves. The leaf surface was seeded with spores, which were assigned a probability of hyphal emergence in a given time interval. Hyphal growth rate was estimated from images obtained in confocal work. Growth was then described using a distribution of angles (where 0° = straight forward) at each step of the algorithm, based on a probability of changing direction again estimated from images. Branching probability was similarly estimated. If a growing hypha encountered a plant cell wall, it was assigned a probability of following the cell wall and then of ceasing to do so, also estimated from images. The probability of a hypha penetrating an encountered stomatal aperture was set to that measured by Shetty et al. (2003). The growth described by this model is random with respect to the leaf surface topography. Thus, the rates of penetration generated by the model are expected to diverge from those observed *in planta* only if the hypothesis of random growth is false.

### 2.9 Humidity experiments

For humidity experiments, inoculation methods and culture conditions were as described above, except that two growth chamber humidities were used (65 and 80% RH). Within each of these, trays of plants were either stood in perspex trays containing 2.5 cm of water, or on wire shelves. At 80% RH, plants on either shelving type were either cloched or left open. This created six humidity regimes. These were: 1, 65% RH; 2, 65% RH + cloche; 3, 80% RH; 4, 80% RH + standing water; 5, 80% RH + cloche; 6, 80% RH + standing water + cloche. Humidities measured at 12 noon among wheat leaves at 15 dpi were: 67%, 70%, 81%, 88%, 93%, 100% (for #1 – #6, respectively). These numbers may have varied over the course of the experiments as the plants grew, but should not have altered their relative position on the scale from lowest to highest, so humidity regimes are referred to simply as #1–#6. Plants were maintained under these humidity regimes throughout the experiment. Confocal microscopy was carried out to determine hyphal growth and stomatal penetration as described above; a minimum of fifty fungi, spread over at least five fields of view, were assessed per humidity regime per day and the whole experiment carried out in three independent repeats.

### 2.10 Wounding experiments

Methods used were the same as those used in the humidity experiment, except that a randomly chosen 50% of wheat plants were gently abraded prior to inoculation. Abrasion caused visible leaf damage but no necrosis or chlorosis. In these experiments, leaves were assessed every three days from 14 dpi onwards for the presence of pycnidia. A minimum of two leaves on each of three plants per wounding/humidity treatment combination were assessed. At 21 dpi, inoculated leaves were harvested and area and number of pycnidia measured in ImageJ (Abramoff et al., 2004) according to the methods described in Fones et al., 2015. The entire experiment was carried out twice independently.

### 2.11 Statistical analyses

Unless otherwise stated in the relevant figure legend, data presented represent means of three or more independent experiments, and error bars show standard error of the mean (SE). For certain experiments, multiple pairwise comparisons were carried out; Bonferroni corrected *t*-tests were used in these instances (Fig. 2, Fig. 3, Fig. 5). Otherwise, Analysis of Variance tests were used on complete datasets, with Tukey’s simultaneous comparisons where appropriate (Fig. 4, Fig. 6, Fig. 7, Fig. 8).

## 3 Results

### 3.1 *Zymoseptoria tritici* germinates rapidly on wheat leaves to form hyphae, but may remain on the leaf surface for several days

In order to quantify the behaviour of *Zymoseptoria tritici* IPO323 on wheat, inoculated leaves were visualised daily using confocal microscopy. By 24 hpi, around 20% of spores on the leaf surface had developed hyphae, with this percentage rising rapidly over the first few days to reach around 75% by 5 dpi (Fig. 1a). Throughout the next seven days, this percentage was unchanged, indicating either 25% permanently non-hyphal individuals or replenishment of non-hyphal individuals through micro-cycle conidiation. Insets in Fig. 1a shows example growth forms. In addition, fungal individuals (as defined in Methods) were scored according to whether any part of the individual had entered the leaf. An average of >99% of individuals were found entirely on the surface until 10 dpi (96%), falling to 60% by 12 dpi (Fig. 1b). Location was confirmed for each fungal individual through careful inspection of z-stacks of confocal images, including 3D reconstructions of z-stacks, allowing tilting of the images, as well as depth-coding of the fungus and the leaf surface / internal structures (see insets to Fig. 1b).

**Supplementary Fig. S1:**
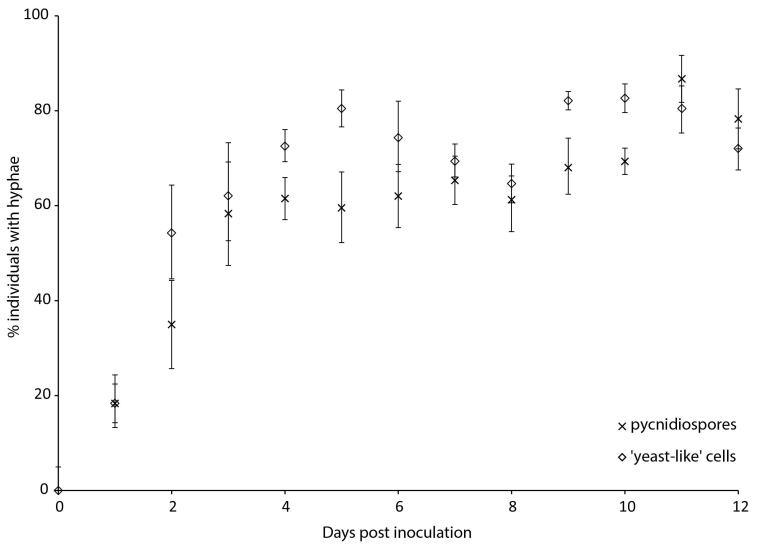


**Supplementary Fig. S2:**
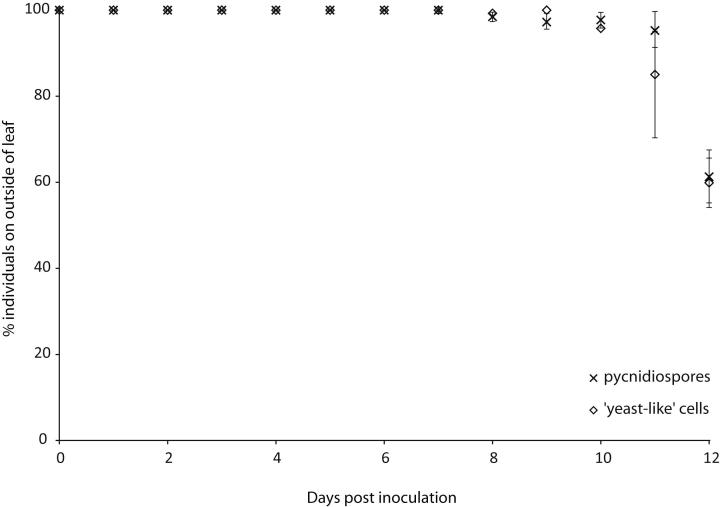


**Supplementary Fig. S3:**
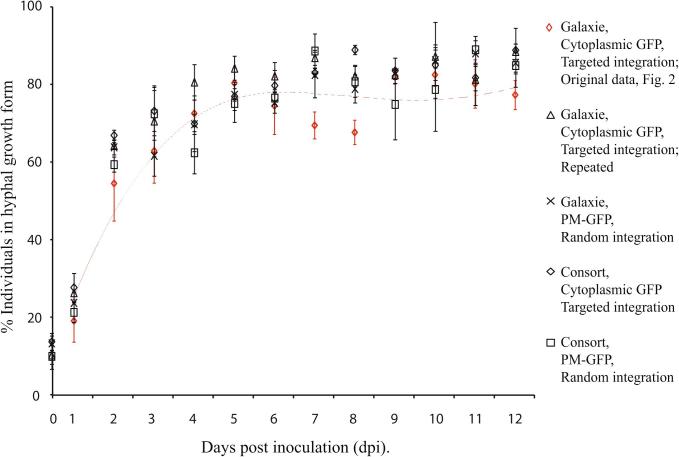


**Supplementary Fig. S4:**
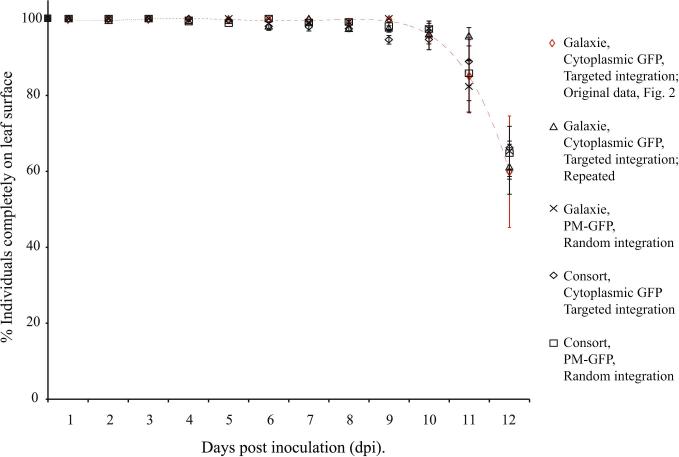


For both hyphal emergence and penetration of the leaf, no differences were detected between yeast-like (plate-raised) inoculum and pycnidiospore inoculum (Figs. S1 and S2). No differences were found between the two differently GFP-tagged strains, or Galaxie *vs.* Consort wheat, with daily germination and penetration rates comparable in all four cases (see Figs. S3 and S4).

### 3.2 Epiphytic *Zymoseptoria tritici* is susceptible to topical fungicide treatment and involved in disease

The data in Figs. 1, S2 and S4 show that at 7 dpi, almost 100% of fungi remain epiphytic, while by 12 dpi, 40–50% have penetrated the leaf. To confirm that the observed epiphytic fungi have a role in disease, inoculated plants were sprayed with fungicide (avoiding runoff) at 7 and 14 dpi. Two fungicides were used: carbendazim, active on the leaf surface (FAO, 1992), and phosphite, a fungitoxin also able to elicit plant defences (Liu et al., 2016). Controls were either water only (avoiding runoff) or no spray at all. There were no significant differences between water applied at 7 dpi or 14 dpi and unsprayed controls. Compared to water controls, phosphite was highly effective in reducing pycnidial numbers whether applied at 7 or 14 dpi, while carbendazim was ineffectual (no significant reduction in pycnidial numbers compared to no-fungicide controls) if applied at 14 dpi (Fig. 2), despite more than halving pycnidial numbers if applied at 7 dpi. This indicates that epiphytic fungi, accessible to carbendazim, remain important for disease at 7, but not 14, dpi.

### 3.3 *Zymoseptoria tritici* does not attach firmly to the leaf and is easily dislodged by water

We hypothesised that the use of a GFP-tagged strain may have allowed us to visualise *Z. tritici* hyphae and spores which would have been washed from the leaf by more invasive methods, such as tissue staining or preparation of samples for electron microscopy. After such procedures, individuals that had penetrated the leaf might form a disproportionate fraction of the remaining fungus, causing underestimation of the average time to penetration.

This hypothesis requires *Z. tritici* to be easily dislodged from the leaf. This was tested by enumerating spores in a fixed area of leaf after various degrees of washing: leaves were either unwashed, misted with a fine water spray to runoff, rinsed in water, or vigorously shaken in water or 0.1% (v/v) non-ionic surfactant Tween-20. Fungi were scored for association with trichomes, encounters with stomata or stomatal apertures and penetration of stomata (Fig. 3). On the day of inoculation, around 50% of *Z. tritici* could be detached from the leaf by misting. This percentage was higher for rinsing and for shaking in water, when only 30% remained. When 0.1% (v/v) Tween was used, less than 10% of control numbers remained. The attachment of *Z. tritici* to the leaf was not much greater after ten days’ (pre-washing) growth, with around 40% removed by misting. At both time points, washed leaf samples showed a significantly greater proportion of trichome-associated *Z. tritici* individuals than control, suggesting that trichomes aid adhesion. A significant increase in the percentage of fungi penetrating stomata could be seen at 10 dpi in samples shaken in water; penetration appeared more common after non-penetrating fungi had been washed from the leaf.

### 3.4 Examples of early penetration can be found at low frequency and are easier to locate in rinsed leaf samples

We hypothesised that *Z. tritici* grows randomly on the leaf surface, finding and entering stomata according to an approximately Poisson distribution in time. If so, active searching might allow examples of early penetration to be discovered, although the probability of finding penetration events would increase with time. In combination with the results shown in Fig. 3, a significant bias towards discovery of penetration events at all time points may then have occurred in earlier works, despite scientific rigour with respect to the questions they were designed to study. Therefore, inoculated control and inoculated rinsed leaves were visualised and hundreds of *Z. tritici* scored for penetration of the leaf in these samples. The results of this experiment are shown in Fig. 4. Examples of penetration can be discovered as early as 1 dpi, and numbers increase with time. In rinsed leaves, from which approximately half of the non-penetrating fungus has been dislodged (Fig. 3), the rate of discovery of penetration events is roughly double that in control samples. Both datasets can be described by an exponential curve with R^2^ value >95%.

### 3.5 The observed pattern of stomatal penetration events over time is consistent with the results of a simulation based upon random fungal growth on the leaf surface

A low rate of penetration from 1 dpi onwards is consistent with the hypothesis of random growth over the leaf surface by *Z. tritici*. To test this hypothesis, we developed a simple algorithm to model the growth of *Z. tritici* on a simplified leaf surface. The model assumes random growth, with parameters from images or literature (Shetty et al., 2003). Examples of the resulting fungal trajectories are shown in Fig. S5. The number of stomatal penetration events was recorded for an initial population size of 1000 individuals and compared to the three penetration time courses of which the average is the control data in Fig. 4. Fig. 5 shows the actual and simulated data; it is clear that the model captures the behaviour of the fungus. Thus, random growth is consistent with the observed rate of stomatal penetration events and the model does not require extra complexity, such as trophic growth towards stomata.

### 3.6 Humidity promotes hyphal growth, stomatal penetration and pycnidiation in *Z. Tritici*

To test the hypothesis that stomatal penetration is promoted at high humidity, further explaining the discrepancy between the current model of *Z .tritici*–wheat interactions and the present results, confocal time courses were repeated with inoculated plants kept under six humidity regimes. As before, the proportion of fungal individuals with hyphae (Fig. 6a) and the proportion of individuals entirely on the leaf surface (Fig. 6b) were determined. Hyphal emergence was promoted, and the percentage of individuals inside the leaf on days 10 and 15 was increased, by high humidity. The number of pycnidia per cm^2^ of inoculated leaf increased with humidity (Fig. 7).

### 3.7 Wounding of the host promotes pycnidiation and shortens the infection cycle

Findings reported herein indicate that *Z. tritici* IPO323 enters the leaf at random after what may be a protracted period of random, epiphytic growth. During this time, humidity favours fungal growth and plant infection. Random growth implies that encountering and penetrating stomata is also random. Wounded leaves, presenting more lesions in the cuticle and epidermis, would thus be more vulnerable to *Z. tritici* infection. We therefore compared the time taken for the first pycnidia to appear, as well as the final density of pycnidia, on gently abraded and control leaves under each of the above (see Fig. 6, Fig. 7) humidity regimes. Wounding promotes pycnidiation and reduces the time required for pycnidiation at all humidities, interacts significantly with humidity in promoting pycnidiation (Fig. 8), as expected under the hypothesis of random fungal growth (Fig. 8).

## 4 Discussion

The results presented here indicate that *Zymoseptoria tritici* is capable of epiphytic growth on susceptible wheat leaves, remaining on the leaf surface for ten days or longer. We did not generally observe internal hyphae before 10 dpi; on average,>95% of individual fungi remained entirely on the leaf surface throughout this period. Supporting this, we found that both phosphite and carbendazim, a plant defence-eliciting and a surface-acting fungicide, respectively, were able to reduce *Z. tritici* disease if applied at 7dpi. The activity of carbendazim at this time point implies that significant amounts of the fungus remain on the surface, as seen in our microscopic studies. By contrast, carbendazim is unable to reduce disease at 14 dpi, when the fungus has mainly entered the leaf. We recognise that the current study may be influenced by growth chamber conditions, and that examination of epiphytic growth under field conditions will be of interest; in addition, it will be interesting to determine whether fungi derived from ascospores show the same pattern of growth.

We hypothesised that the discrepancy between these observations and previous reports of much earlier penetration (Kema et al., 1996, Duncan and Howard, 2000, Cousin et al., 2006, Keon et al., 2007) might be due to (i) the use of a GFP-tagged strain, allowing non-invasive visualisation and giving a more accurate picture, including many non-penetrating fungi which could be easily dislodged during sample preparation or staining for alternative imaging techniques and (ii) the omission, in the present study, of very high humidity/misting and low light/darkness used to determine the manner of fungal ingress into the host (Kema et al., 1996, Duncan and Howard, 2000, Cousin et al., 2006). Further, we hypothesised (iii) that random growth by *Z. tritici* hyphae with respect to the leaf surface, as suspected by Kema et al. (1996), might give rise to a random pattern of stomatal penetration which would allow early penetration events to be discovered if actively sought.

Firstly, we found that the fungi could indeed be readily dislodged from the leaf by the gentle means of misting the leaves with sufficient water to cause runoff. Preparation of samples for visualisation may involve multiple, harsher rinsing steps (staining) or plunging of the sample into liquid nitrogen slush (cryo-SEM), processes obviated here by the use of GFP tagged strains. We showed that the likelihood of detecting penetration events was almost doubled if the leaves had been rinsed prior to visualisation. An unconscious bias in favour of the observation of penetration is thus introduced when that observation depends upon disruptive techniques. Biologically, this facility for being dislodged, even by a water mist similar to fine drizzle, shows that *Z. tritici* is well adapted to rain-splash dispersal, so that if fresh inoculum arrives in a field or if microcycle conidiation occurs on the surface of an infected host, the resulting spores spread easily. Notably, epidemiological studies have identified continuous leaf wetness, rather than heavy rain, as an important risk factor in the spread of this disease (Shaner and Finney, 1976, Shearer and Zadoks, 1972, Shaw and Royle, 1993, Pietravalle et al., 2003).

Following inoculation of a wheat leaf, *Z. tritici* rapidly produces hyphae, which are essential for infection (Cousin et al., 2006, Rudd et al., 2015). Filamentous growth in dimorphic fungi is frequently associated with low nutritional status, with hyphae used to forage for nutrients (Markham, 1992, Biswas and Morschhäuser, 2005). On the leaf surface few nutrients are available, so this foraging growth form may be adopted. Many plant pathogenic fungi are able to detect and respond to a host leaf surface, recognising its hydrophobicity (e.g. *Magnaporthe oryzae* Talbot, 2003), physical features such as the height and spacing of leaf ridges or chemical signals such as leaf alcohols produced by the susceptible host (e.g. *Puccinia graminis*; Read et al., 1997, Collins et al., 2001). By contrast, Kema et al. (1996) noted that hyphae of *Z. tritici* could be frequently seen crossing stomata without penetration, and proposed this as evidence for random growth over the leaf surface, without chemotropic or mechano-sensory growth responses for seeking out stomata. Random growth, in the absence of any other factors controlling penetration, would lead to a temporal distribution of stomatal penetration events which can be approximated by the Poisson distribution. Under this hypothesis it is therefore expected that if enough individuals are observed, penetration will be detected early, with the chance of detecting penetration rising over time. Detections per day would therefore be expected to follow some exponential curve during the initial epiphytic growth period. The function describing this curve is expected to be multiplied, after washing – and thus after staining – by a constant reflecting the proportion of non-penetrating fungal individuals detached by that process. Using sample sizes of hundreds, we have been able to show that such curves are produced by a researcher actively searching for penetration events, and that the percentage of fungi observed to penetrate the leaf is approximately doubled following rinsing in water, which we showed removes 50% of the non-penetrating individuals. Penetrations per day observed on unrinsed leaves agreed well with the daily penetration rates in the output of a model in which fungal growth was random with respect to stomata. There is an apparent, though non-significant, divergence between actual and simulated penetration rates at 9 and 10 dpi, with the random model outperforming the real fungus in entering stomata; this is to be expected, given that the model takes no account of possible fungal death or reduced growth rates due to prolonged survival on the leaf surface, nor of potential plant defences induced by early-penetrating fungi which may make later penetration more difficult. Together, these data support the hypothesis of random growth and our hypothesis that the discrepancy between our results and previously reported data concerning time of penetration is due, or partially due, to the previous reliance upon staining methods rather than GFP-tagged *Z. tritici*.

We also tested the hypothesis that part of the discrepancy between our observed time to penetration and that previously reported was caused by our choice of incubation conditions, not optimised for penetration. We found that humidity affects the epiphytic growth form adopted by *Z. tritici*, promoting the hyphal form, which is more adept at covering distance across the leaf surface. If *Z. tritici* grows randomly across the leaf rather than seeking stomata, we expect this increased hyphal growth to have a marked effect upon the time taken to achieve ingress. We note that it might be argued that the findings of random fungal growth reported here are artefacts of high humidity growth chamber conditions that mask signals normally used by *Zymoseptoria* to detect stomata. However, we show that increasing humidity reduces the time taken for penetration. In addition, we show that the number of pycnidia per leaf increases with humidity. Both results are evidence that the observed increase in hyphal growth at high humidity has the consequences expected under the random growth hypothesis. High humidity, far from preventing stomatal detection, promoted stomatal penetration. This is in line with findings reported by Kema et al. (1996), who found that inoculated leaves required high humidity for at least 48 hpi for infection to be established. Further, epidemiological studies have identified prolonged leaf wetness as the greatest risk factor for Septoria blotch disease (Shaner and Finney, 1976, Shaw and Royle, 1993, Shaw and Royle, 1993, Chungu et al., 2001, Pietravalle et al., 2003), with the fungus unable to cause disease under very warm conditions or during prolonged dry spells (Savary et al., 2015). These data are supported by the importance of leaf wetness in predicting outbreaks of Septoria blotch disease in wheat (Markham, 1992, Biswas and Morschhäuser, 2005).

Finally, on wounding the wheat leaves, we found that more pycnidia developed faster, at all humidities. At lower humidities, when the leaves stay wet for less time, the fungus is more likely to be exposed to drought stress before it finds a stoma. With random growth and entry by *Z. tritici*, having wounds in the epidermis reduces the average time taken for the fungus to achieve entry. This means drying stresses are minimised and the fungus is more likely to enter the plant, at random, through this increased number of epidermal lesions, before the lack of moisture becomes critical. Thus, successful infection is more likely on a wounded plant. Conversely, on unwounded plants at high humidity, fungal filamentation is promoted, along with increased stomatal opening, both of which may promote rapid, successful infection.

The findings concerning wounded plants have other implications. Firstly, they suggest that stomata are not necessary for fungal ingress. This may increase the risk from *Z. tritici* following high winds, hail, or other leaf damaging weather. For winter and spring crops in the UK, this could be a significant issue in the face of climate change-induced increases in severe weather events including Atlantic storms during the growing season (Woollings et al., 2012). In addition, this suggests that any wheat varieties with a higher density of stomata might be more vulnerable to infection.

Random, epiphytic growth of *Z. tritici* is both a threat and an opportunity. On the one hand, an epiphytic population which is capable of budding growth provides a large reservoir of infective material to be rain-splashed throughout the crop and on to volunteers and other species. The finding that *Z. tritici* can survive without entering the host for up to ten days raises the possibility that *Z. tritici* may easily persist away from suitable hosts. On the other hand, epiphytic growth represents a period in the fungal lifecycle when it is not protected by the host and may be more vulnerable to chemical control.

The results presented here also have implications for the way in which we think about the asexual lifecycle of *Z. tritici* and its interaction with wheat. The capacity of this fungus to remain epiphytic casts a new light in which to interpret genomic, transcriptomic, and proteomic studies. Indeed, the symptomless phase of *Z. tritici* infection may comprise considerably less internal fungus than previously thought. This may help to explain why the fungus does not produce biotrophic feeding structures within the host and secretes mainly cutinases, peptidases and lipases during early infection (Rudd et al., 2015, Palma-Guerrero et al., 2016). In addition, the lack of symptoms, the unchanging composition of the wheat apoplast (Keon et al., 2007) and the ability of *Z. tritici* to evade the host’s defences in this period might be simply explained by the fact that little or no fungus is inside the leaf during the first few days. This would be in line with the ‘latent necrotrophy’ postulated by Sánchez-Vallet et al. (2015) as the best description of the infection behaviour of *Z. tritici*. The switch to necrotrophic feeding, exponential increase in fungal biomass and onset of plant defence responses such as programmed cell death (PCD) from around 10 dpi onwards might be triggered by the increased fungal ingress at this time. Of course, it has been established that wheat begins to respond to *Z. tritici* early in infection, with changes in gene expression seen preceding even the earliest published penetration times (Ray et al., 2003, Adhikari et al., 2007, Orton et al., 2011). Thus, observed plant defence responses do not necessarily indicate extensive fungal ingress. Similarly, the pathogen is known to produce LysM proteins, thought to protect the fungus from plant recognition, from 4 dpi, but LysM production is much higher by 9 dpi (Marshall et al., 2011), and is thus not irreconcilable with a high proportion of fungus remaining epiphytic during the symptomless phase. LysM may be produced either in proportion to penetration (higher at day 9 than day 4 with random growth) or to shield epiphytic fungus from the observed pre-penetration defences.

A final implication of these results is that the epiphytic growth may be a remnant of a pre-pathogenic lifestyle, potentially yielding clues to the evolution of *Z. tritici*. It would be interesting to determine whether the degree of epiphytic growth and the timing of entry vary between strains of *Z. tritici*, and whether such variation is reflected in the genomes of the strains and in comparison to fungi that show a purely epiphytic lifestyle.

## Author contributions

HF conceived, designed and carried out experiments, analysed images and data and wrote the manuscript. CE wrote the computer simulation of *Z. tritici* growth. JC carried out preliminary work concerning epiphytic growth of *Z. tritici.* WK conducted the fungicide experiments*.* SG oversaw the project, reviewed the manuscript and obtained funding.

## Competing interests

The authors have no competing interests.

## Funding

HF, CE, WK and SG were funded by BBSRC grant: and JC by a BSPP summer studentship.

## Figures and Tables

**Supplementary Fig. S5:**