# Supplementary material for: A role for random, humidity-dependent epiphytic growth prior to invasion of wheat by Zymoseptoria tritici
Source: Fungal Genet Biol. 2017 Sep;106:51–60. doi: 10.1016/j.fgb.2017.07.002 (PMC5556705; doi:10.1016/j.fgb.2017.07.002)
Supplement: Supplementary data 1 [file mmc1.docx]

**Supplementary Figure Legends**

**Figure S1: Comparison of the proportion of *Z. tritici* individuals showing hyphal growth during the first 12 days following inoculation of lab-grown (yeast-like cells; diamonds) *vs.* true pycnidiospores (crosses) of IPO323 onto Galaxie wheat leaves.** A minimum of thirty fungal individuals were photographed on each day and resulting images scored for hyphal growth on days one to twelve. This time-course was carried out on six independent occasions for lab-grown fungus and two for pycnidiospores. Values are means of these independent experiments and error bars show SE.

**Figure S2: Comparison of the proportion of *Z. tritici* individuals growing exclusively on the leaf surface during the first 12 days following inoculation of lab-grown (yeast-like cells; diamonds) *vs.* true pycnidiospores (crosses) of IPO323 onto Galaxie wheat leaves.** A minimum of thirty fungal individuals were photographed on each day and resulting images scored for external *vs.* internal growth on days one to twelve. This time-course was carried out on six independent occasions for lab-grown fungus and two for pycnidiospores. Values are means of these independent experiments and error bars show SE.

**Figure S3: Comparison of the proportion of *Z. tritici* individuals showing hyphal growth during the first 12 days following inoculation of two different GFP-tagged IPO323 strains onto Galaxie and Consort wheat leaves.** A minimum of seventy-five fungal individuals on each of three independent samples were scored for hyphal growth on days one to twelve. Original results from Figure 1 (Galaxie, cytoplasmic GFP) are shown in red for comparison. This combination is repeated here (black triangles) along with the same strain on Consort (black diamonds) and the second, randomly integrated, plasma membrane labelled, GFP tagged strain on Galaxie (black X’s) and Consort (black squares). Values are means of the independent sample data and error bars show SE (n = 3).

**Figure S4: Comparison of the proportion of *Z. tritici* individuals growing exclusively on the leaf surface during the first 12 days following inoculation of two different GFP-tagged IPO323 strains onto Galaxie and Consort wheat leaves.** A minimum of seventy-five fungal individuals on each of three independent samples were scored for external *vs.* internal growth on days one to twelve. Values are means of the independent sample data and error bars show SE (n = 3). Original results from Figure 2 (Galaxie, cytoplasmic GFP) are shown in red for comparison. This combination is repeated here (black triangles) along with the same strain on Consort (black diamonds) and the second, randomly integrated, plasma membrane labelled, GFP tagged strain on Galaxie (black X’s) and Consort (black squares). Values are means of the independent sample data and error bars show SE (n = 3).

**Figure S5: Example trajectories of modelled fungal growth.**
